# Left Bundle Branch Area Pacing over His Bundle Pacing: How Far Have We Come?

**DOI:** 10.3390/jcm12093251

**Published:** 2023-05-02

**Authors:** Matteo Baroni, Alberto Preda, Marisa Varrenti, Sara Vargiu, Marco Carbonaro, Federica Giordano, Lorenzo Gigli, Patrizio Mazzone

**Affiliations:** Cardio-Thoraco-Vascular Department, Electrophysiology Unit, ASST Grande Ospedale Metropolitano Niguarda, 20162 Milan, Italy; matteo.baroni@ospedaleniguarda.it (M.B.); marisa.varrenti@ospedaleniguarda.it (M.V.); sara.vargiu@ospedaleniguarda.it (S.V.); marco.carbonaro@ospedaleniguarda.it (M.C.); federica.giordano@ospedaleniguarda.it (F.G.); lorenzo.gigli@ospedaleniguarda.it (L.G.); patrizio.mazzone@ospedaleniguarda.it (P.M.)

Implantable cardiac pacemakers have greatly evolved during the few past years, focusing on newer modalities of physiologic cardiac pacing. Indeed, non-physiologic pacing modalities, such as right ventricular (RV) pacing, have been shown to cause systolic dysfunction and heart failure (HF) over time. On the contrary, preserving myocardial synchrony during systole showed not only to improve cardiac contraction through myocardial reverse remodeling [1] but also to restore the native electrical conduction system [2]. These benefits have been demonstrated in elderly patients too [3]. Conduction system pacing (CSP) encompassing His bundle (HBP) and left bundle branch area pacing (LBBAP) are relatively novel modalities that have the potential to outperform conventional pacing approaches [4]. Although data relative to clinical endpoints is currently limited, CSP is proving to be a reliable alternative to cardiac resynchronization therapy (CRT) [5]. HBP represents the most physiological form of cardiac stimulation. Compared to conventional RV pacing, HBP decreases interventricular dyssynchrony and reduces mitral and tricuspid valve regurgitation [6,7]. Clinically, HBP was demonstrated to improve NYHA functional class and LV ejection fraction (LVEF) in CRT candidates, while a reduction of a combined endpoint of mortality and HF hospitalizations during long-term follow-up was reported in nonrandomized registry studies [8,9]. Moreover, HBP was equivalent or even superior to CRT with respect to QRS narrowing in two randomized trials [10,11]. Despite all the benefits provided by HPS, several practical concerns limit its efficacy, including the bundle branch correction rate and electrical lead performance over time [12,13,14]. Compared to HBP, data on LBBAP are even more scarce and largely based on smaller registry studies. Nevertheless, LBBAP provides significantly lower and more stable capture thresholds over the long term compared to HPS [15]. QRS complex shortening is usually achieved, and full correction of the left bundle branch block can more often and more easily be obtained. Furthermore, the excellent sensing makes LBBAP leads suitable for sensing in implantable cardioverter defibrillators (ICDs), as was demonstrated in the CROSS-LEFT pilot study [16]. Novel studies comparing LBBAP with CRT reported a higher rate of cardiac reverse remodeling associated with improvement of LVEF in LBBAP patients, with consensual improvement of functional status and clinical outcomes [17,18]. According to the prospective randomized LBBP-RESYNC trial, in 40 patients with non-ischemic cardiomyopathy and left bundle branch block with CRT indication, LBBAP provided greater improvement in the LVEF compared to conventional CRT even in the intention-to-treat analysis [19]. Positive effects on LVEF are also reported in patients with right bundle branch block, HF, and mildly reduced LVEF [4]. Success rates of LBBAP lead implantation attempts are reported to be >90% for conventional bradycardia indications and >80% for HF [20]. The introduction of dedicated delivery systems has substantially improved operators performance. New delivery sheaths with different lengths, curves, and braided tubes for variable stiffness along the sheath body are available today [21]. The limits and possible risks of this procedure are relatively few and acceptable. The implantation duration and fluoroscopy time still remain slightly longer compared to conventional RV lead implantation [22]. Iodinated contrast media are needed to perform septography. A lead position deep in the interventricular septum might raise concerns regarding the long-term extractability of LBBAP leads [23]. Overall, all the above-mentioned studies favor LBBAP over HPS as an alternative to CRT. These data are further supported by two studies that explore the use of LBBAP as a bailout strategy in the case of failed coronary sinus lead implantation in CRT candidates [24]. Even if current published studies investigating LBBAP are often monocentric, retrospective, and descriptive in nature, comforting data have been provided, leading to the initiation of a number of newer trials. Among these, the multicentric prospective randomized controlled PROTECT-SYNC (NCT05585411) aims to randomize 450 patients with bradycardia pacing indications who require substantial (>40%) ventricular pacing to either RV pacing or LBBAP with the primary composite endpoint of death, HF hospitalizations, and upgrade to CRT. The LEAP-BLOCK study (NCT04730921) is a randomized controlled trial designed to investigate whether LBBAP reduces the risk of RV-pacing-induced cardiac dysfunction compared to conventional pacing in patients with an AV block and normal LV function. Finally, more and more interest is aroused in the possibility of using LBBAP in patients with an indication for anti-bradycardia pacing after transcatheter aortic valve replacement (TAVR) [25]. Indeed, new onset atrioventricular conduction block remains a frequent complication of TAVR. Since standard RV pacing at high pacing burden may lead to deterioration of LVEF, LBBAP might be an advantageous solution for this category of individuals. Novel data from randomized studies are expected (NCT05024279, NCT05541679).
